# The role of data science in healthcare advancements: applications, benefits, and future prospects

**DOI:** 10.1007/s11845-021-02730-z

**Published:** 2021-08-16

**Authors:** Sri Venkat Gunturi Subrahmanya, Dasharathraj K. Shetty, Vathsala Patil, B. M. Zeeshan Hameed, Rahul Paul, Komal Smriti, Nithesh Naik, Bhaskar K. Somani

**Affiliations:** 1 Department of Electrical and Electronics Engineering, Manipal Institute of Technology, Manipal Academy of Higher Education, Manipal, Karnataka India; 2 Department of Humanities and Management, Manipal Institute of Technology, Manipal Academy of Higher Education, Manipal, Karnataka India; 3 Department of Oral Medicine and Radiology, Manipal College of Dental Sciences, Manipal, Manipal Academy of Higher Education, Manipal Karnataka, India; 4 Department of Urology, Father Muller Medical College, Mangalore, Karnataka India; 5 Department of Radiation Oncology, Massachusetts General Hospital, Boston, MA USA; 6 Department of Mechanical and Manufacturing Engineering, Manipal Institute of Technology, Manipal Academy of Higher Education, Manipal, Karnataka India; 7 Department of Urology, University Hospital Southampton NHS Trust, Southampton, UK

**Keywords:** Big data, Data analytics, Data mining, Healthcare, Healthcare informatics

## Abstract

Data science is an interdisciplinary field that extracts knowledge and insights from many structural and unstructured data, using scientific methods, data mining techniques, machine-learning algorithms, and big data. The healthcare industry generates large datasets of useful information on patient demography, treatment plans, results of medical examinations, insurance, etc. The data collected from the Internet of Things (IoT) devices attract the attention of data scientists. Data science provides aid to process, manage, analyze, and assimilate the large quantities of fragmented, structured, and unstructured data created by healthcare systems. This data requires effective management and analysis to acquire factual results. The process of data cleansing, data mining, data preparation, and data analysis used in healthcare applications is reviewed and discussed in the article. The article provides an insight into the status and prospects of big data analytics in healthcare, highlights the advantages, describes the frameworks and techniques used, briefs about the challenges faced currently, and discusses viable solutions. Data science and big data analytics can provide practical insights and aid in the decision-making of strategic decisions concerning the health system. It helps build a comprehensive view of patients, consumers, and clinicians. Data-driven decision-making opens up new possibilities to boost healthcare quality.

## Introduction

The evolution in the digital era has led to the confluence of healthcare and technology resulting in the emergence of newer data-related applications [1]. Due to the voluminous amounts of clinical data generated from the health care sector like the Electronic Health Records (EHR) of patients, prescriptions, clinical reports, information about the purchase of medicines, medical insurance-related data, investigations, and laboratory reports, there lies an immense opportunity to analyze and study these using recent technologies [2]. The huge volume of data can be pooled together and analyzed effectively using machine-learning algorithms. Analyzing the details and understanding the patterns in the data can help in better decision-making resulting in a better quality of patient care. It can aid to understand the trends to improvise the outcome of medical care, life expectancy, early detection, and identification of disease at an initial stage and required treatment at an affordable cost [3]. Health Information Exchange (HIE) can be implemented which will help in extracting clinical information across various distinct repositories and merge it into a single person’s health record allowing all care providers to access it securely. Hence, the organizations associated with healthcare must attempt to procure all the available tools and infrastructure to make use of the big data, which can augment the revenue and profits and can establish better healthcare networks, and stand apart to reap significant benefits [4, 5]. Data mining techniques can create a shift from conventional medical databases to a knowledge-rich, evidence-based healthcare environment in the coming decade.

Big data and its utility in healthcare and medical sciences have become more critical with the dawn of the social media era (platforms such as Facebook and Twitter) and smartphone apps that can monitor personal health parameters using sensors and analyzers [6, 7]. The role of data mining is to improvise the stored user information to provide superior treatment and care. This review article provides an insight into the advantages and methodologies of big data usage in health care systems. It highlights the voluminous data generated in these systems, their qualities, possible security-related problems, data handling, and how this analytics support gaining significant insight into these data set.

## Search strategy

A non-systematic review of all data science, big data in healthcare-related English language literature published in the last decade (2010–2020) was conducted in November 2020 using MEDLINE, Scopus, EMBASE, and Google Scholar. Our search strategy involved creating a search string based on a combination of keywords. They were: “Big Data,” “Big Data Analytics,” “Healthcare,” “Artificial Intelligence,” “AI,” “Machine learning,” “ML,” “ANN,” “Convolutional Networks,” “Electronic Health Records,” “EHR,” “EMR,” “Bioinformatics,” and “Data Science.” We included original articles published in English.

### Inclusion criteria

1. Articles on big data analytics, data science, and AI.
2. Full-text original articles on all aspects of application of data science in medical sciences.

### Exclusion criteria

1. Commentaries, reviews, and articles with no full-text context and book chapters.
2. Animal, laboratory, or cadaveric studies.

The literature review was performed as per the above-mentioned strategy. The evaluation of titles and abstracts, screening, and the full article text was conducted for the chosen articles that satisfied the inclusion criteria. Furthermore, the authors manually reviewed the selected article’s references list to screen for any additional work of interest. The authors resolved the disagreements about eligibility for a consensus decision after discussion.

## Knowing more about “big data”

Big data consists of vast volumes of data, which cannot be managed using conventional technologies. Although there are many ways to define big data, we can consider the one defined by Douglas Laney [8] that represents three dimensions, namely, volume, velocity, and variety (3 Vs). The “big” in big data implies its large volume. Velocity demonstrates the speed or rate at which data is processed. Variety focuses on the various forms of structured and raw data obtained by any method or device, such as transaction-level data, videos, audios, texts, emails, and logs. The 3 Vs became the default description of big data, while many other Vs are added to the definition [9]. “Veracity” remains the most agreed 4th “V.” Data veracity focuses on the accuracy and reliability of a dataset. It helps to filter through what is important and what is not. The data with high veracity has many records that are valuable to analyze and that contribute in a meaningful way to the overall results. This aspect poses the biggest challenge when it comes to big data. With so much data available, ensuring that it is relevant and of high quality is important. Over recent years, big data has become increasingly popular across all parts of the globe.

Big data needs technologically sophisticated applications that use high-end computing resources and Artificial Intelligence (AI)-based algorithms to understand such huge volumes of data. Machine learning (ML) approaches for automatic decision-making by applying fuzzy logic and neural networks will be added advantage. Innovative and efficient strategies for dealing with data, smart cloud-based applications, effective storage, and user-friendly visualization are required for big data to gain practical insights [10].

## Medical care as a repository for big data

Healthcare is a multilayered system developed specifically for preventing, diagnosing, and treating diseases. The key elements of medical care are health practitioners (physicians and nurses), healthcare facilities (which include clinics, drug delivery centers, and other testing or treatment technologies), and a funding agency that funds the former. Health care practitioners belong to different fields of health such as dentistry, pharmacy, medicine, nursing, psychology, allied health sciences, and many more. Depending on the severity of the cases, health care is provided at many levels. In all these stages, health practitioners need different forms of information such as the medical history of the patient (data related to medication and prescriptions), clinical data (such as data from laboratory assessments), and other personal or private medical data. The usual practice for a clinic, hospital, or patient to retain these medical documents would be maintaining either written notes or in the form of printed reports [11].

The clinical case records preserve the incidence and outcome of disease in a person’s body as a tale in the family, and the doctor plays an integral role in this tale [12]. With the emergence of electronic systems and their capacity, digitizing medical exams, health records, and investigations is a common procedure today. In 2003, the Institute of Medicine, a division in the National Academies of Sciences and Engineering coined the term “Electronic Health Records” for representing an electronic portal that saves the records of the patients. Electronic health records (EHRs) are automated medical records of patients related to an individual’s physical/mental health or significant reports that are saved in an electronic system and used to record, send, receive, store, retrieve, and connect the medical personnel and patient with medical services [13].

## Open-source big data platforms

It is an inefficient idea to work with big data or vast volumes of data into storage considering even the most powerful computers. Hence, the only logical approach to process large quantities of big data available in a complex form is by spreading and processing it on several parallel connected nodes. Nevertheless, the volume of the data is typically so high that a large number of computing machines are needed in a reasonable period to distribute and finish processing. Working with thousands of nodes involves coping with issues related to paralleling the computation, spreading of data, and manage failures. Table 1 shows the few open sources of big data platforms and their utilities for data scientists.

**Table 1:** Open source big data platforms and their utilities

Big data tools	Utilities
Apache Hadoop	It is designed to scale up to thousands of machines from single servers, each of which offers local storage The framework enables users to easily build and validate distributed structures, distributes data, and operates across machines automatically
Apache Spark	The Hadoop Distributed File system (HDFS) and other data stores are flexible to work with Spark offers integrated Application Program Interfaces (APIs) which enable users to write apps in different languages
Apache Cassandra	Cassandra is highly flexible and can add additional hardware that can handle more data and users on demand Cassandra adapts to all possible data types such as unstructured, structured, and semi-structured supporting features such as Atomicity, Consistency, Isolation, and Durability (ACID)
Apache Storm	In several cases, Apache Storm is easy to integrate with any programming language, with real-time analytics, online machine learning, and computation Apache Storm uses parallel calculations which run across a machine cluster
RapidMiner	RapidMiner provides a variety of products for a new process of data mining It provides an integrated data preparation environment, machine learning, text mining, visualization, predictive analysis, application development, prototype validation, and implementation. statistic modeling, deployment
Cloudera	Users can spin clusters, terminate them, and only pay for what they need Cloudera Enterprise can be deployed and run on AWS and Google Cloud Platforms by users

## Data mining

**Fig. 1:**
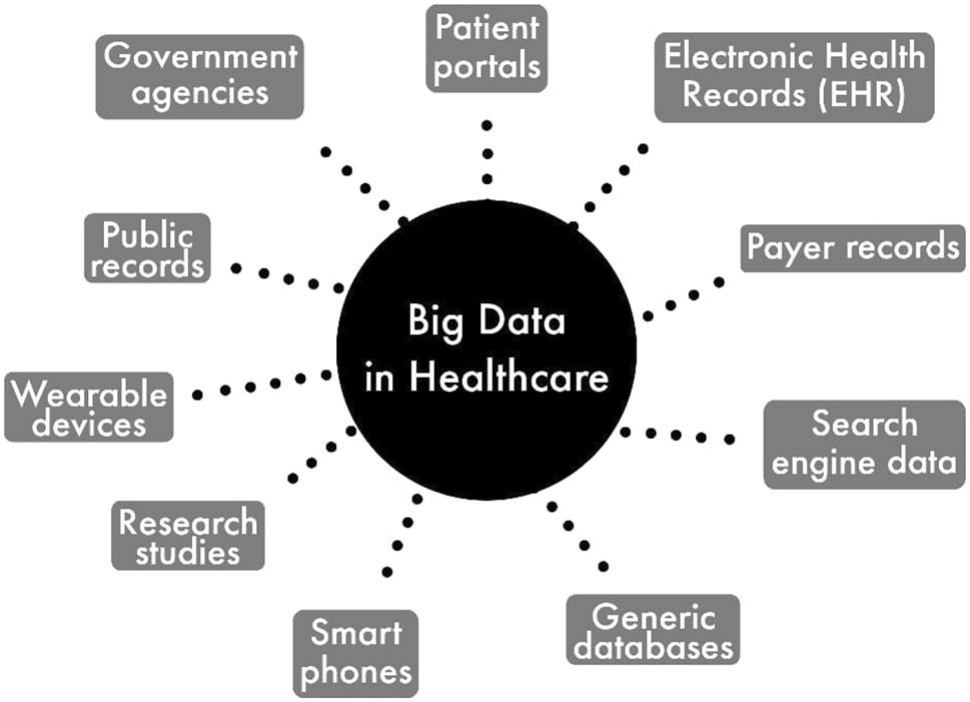
Sources of big data in healthcare

Data types can be classified based on their nature, source, and data collection methods [14]. Data mining techniques include data grouping, data clustering, data correlation, and mining of sequential patterns, regression, and data storage. There are several sources to obtain healthcare-related data (Fig. 1). The most commonly used type (77%) is the data generated by humans (HG data) which includes Electronic Medical Records (EMR), Electronic Health Records (EHR), and Electronic Patient Records (EPR). Online data through Web Service (WS) is considered as the second largest form of data (11%) due to the increase in the number of people using social media day by day and current digital development in the medical sector [15]. Recent advances in the Natural Language Processing (NLP)-based methodologies are also making WS simpler to use [16]. The other data forms such as Sensor Data (SD), Big Transactional Data (BTD), and Biometric Data (BM) make around 12% of overall data use, but wearable personal health monitoring devices’ prominence and market growth [17] may need SD and BM data.

## Applications of analytics in healthcare

**Fig. 2:**
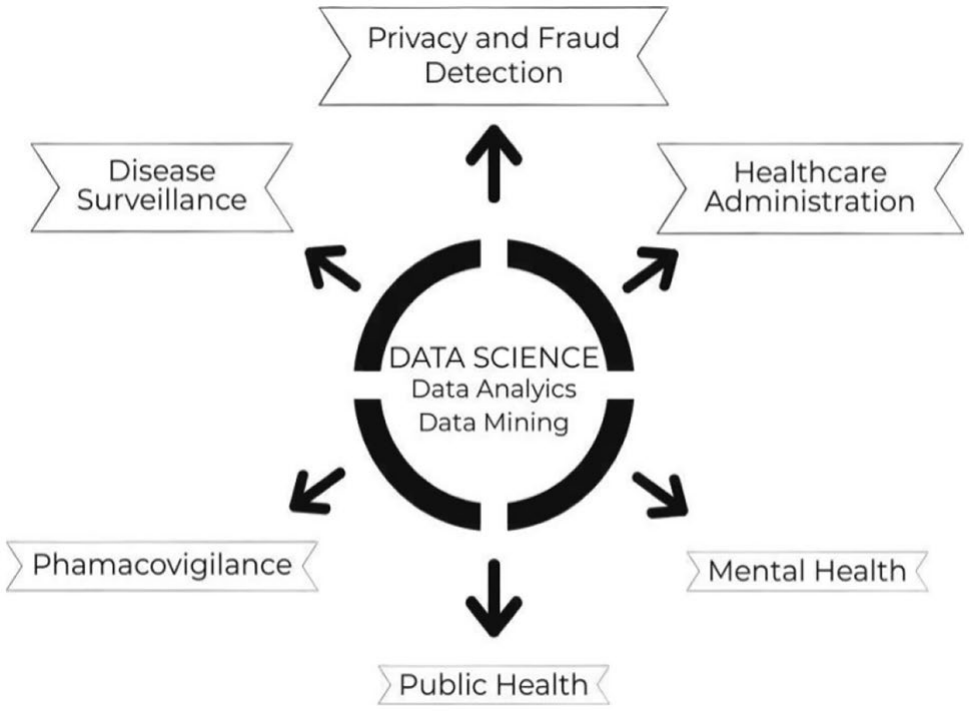
Various applications of data science in healthcare

There are six areas of applications of analytics in healthcare (Fig. 2) including disease surveillance, health care management and administration, privacy protection and fraud detection, mental health, public health, and pharmacovigilance. Researchers have implemented data extraction for data deposition and cloud-based computing, optimizing quality, lowering costs, leveraging resources, handling patients, and other fields.

## Disease surveillance

**Fig. 3:**
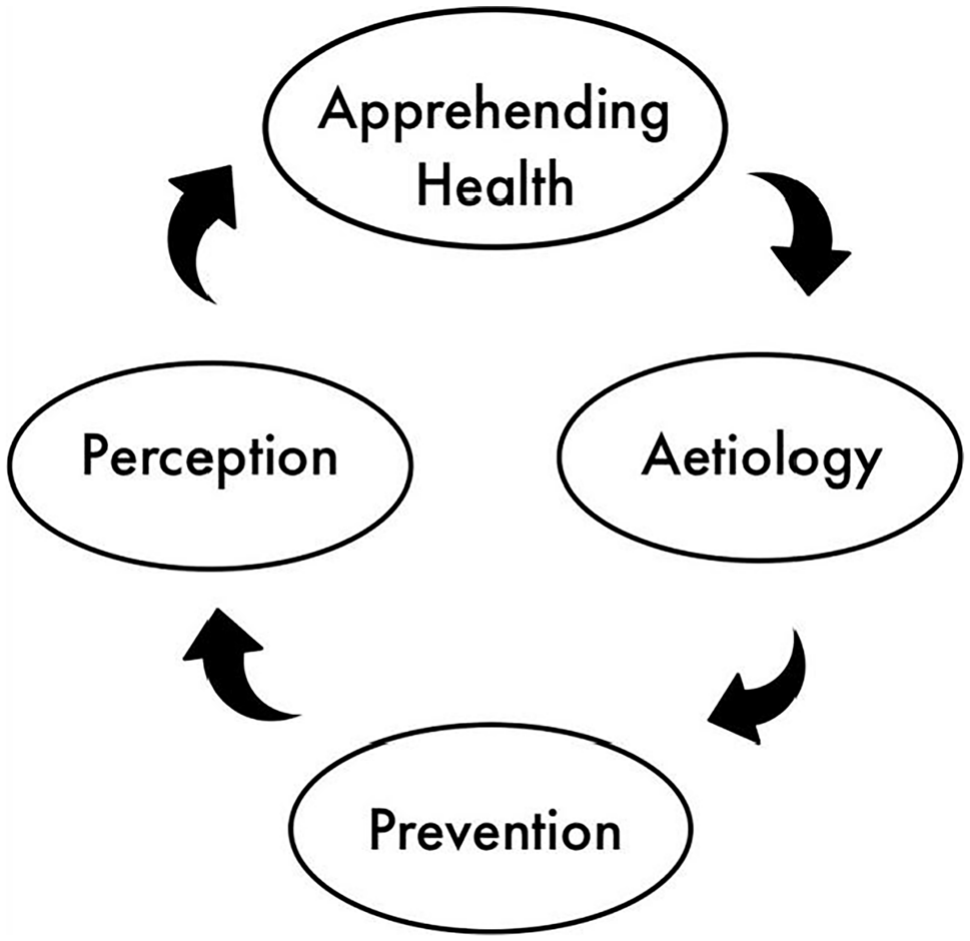
The disease analysis system

It involves the perception of the disease, understanding its condition, etiology (the manner of causation of a disease), and prevention (Fig. 3).

Information obtained with the help of EHRs, and the Internet has a huge prospect for disease analysis. The various surveillance methods would aid the planning of services, evaluation of treatments, priority setting, and the development of health policy and practice.

## Image processing of healthcare data from the big data point of view

Image processing on healthcare data offers valuable knowledge about anatomy and organ functioning and identifies the disease and patient health conditions. The technique currently has been used for organ delineation, identification of lung tumors, diagnosis of spinal deformity, detection of arterial stenosis, detection of an aneurysm, etc. [18]. The wavelets technique is commonly used for image processing techniques such as segmentation, enhancement, and noise reduction. The use of artificial intelligence in image processing will enhance aspects of health care including screening, diagnosis, and prognosis, and integrating medical images with other types of data and genomic data will increase accuracy and facilitate early diagnosis of diseases [18, 19]. The exponential increase in the count of medical facilities and patients has led to better use of clinical settings of computer-based healthcare diagnostics and decision-making systems.

### Data from wearable technology

Multi-National Companies like Apple and Google are working on health-based apps and wearable technology as part of a broader range of electronic sensors, the so-called IoT, and toolkits for healthcare-related apps. The possibility of collecting accurate medical data on real-time (e.g., mood, diet followed, exercise, and sleep cycles patterns), linked to physiological indicators (e.g., heart rate, calories burned, level of blood glucose, cortisol levels), is perhaps discrete and omnipresent at minimum cost, unrelated to traditional health care. “True Colors” is a wearable designed to collect continuous patient-centric data with the accessibility and acceptability needed to allow for accurate longitudinal follow-up. More importantly, this system is presently being piloted as a daily health-monitoring substitute.

### Medical signal analytics

Telemetry and the devices for the monitoring of physiological parameters generate large amounts of data. The data generated generally are retained for a shorter duration, and thus, extensive research into produced data is neglected. However, advancements in data science in the field of healthcare attempt to ensure better management of data and provide enhanced patient care [20–23].

The use of continuous waveform in health records containing information generated through the application of statistical disciplines (e.g., statistical, quantitative, contextual, cognitive, predictive, etc.) can drive comprehensive care decision-making. Data acquisition apart from an ingestion-streaming platform is needed that can control a set of waveforms at various fidelity rates. The integration of this waveform data with the EHR’s static data results in an important component for giving analytics engine situational as well as contextual awareness. Enhancing the data collected by analytics will not just make the method more reliable, but will also help in balancing predictive analytics’ sensitivity and specificity. The signal processing species must mainly rely on the kind of disease population under observation.

Various signal-processing techniques can be used to derive a large number of target properties that are later consumed to provide actionable insight by a pre-trained machine-learning model. Such observations may be analytical, prescriptive, or predictive. Such insights can be furthermore built to activate other techniques such as alarms and physician notifications. Maintaining these continuous waveforms–based data along with specific data obtained from the remaining sources in perfect harmony to find the appropriate patient information to improve diagnosis and treatments of the next generation can be a daunting task [24]. Several technological criteria and specifications at the framework, analytical, and clinical levels need to be planned and implemented for the bedside implementation of these systems into medical setups.

## Healthcare administration

**Fig. 4:**
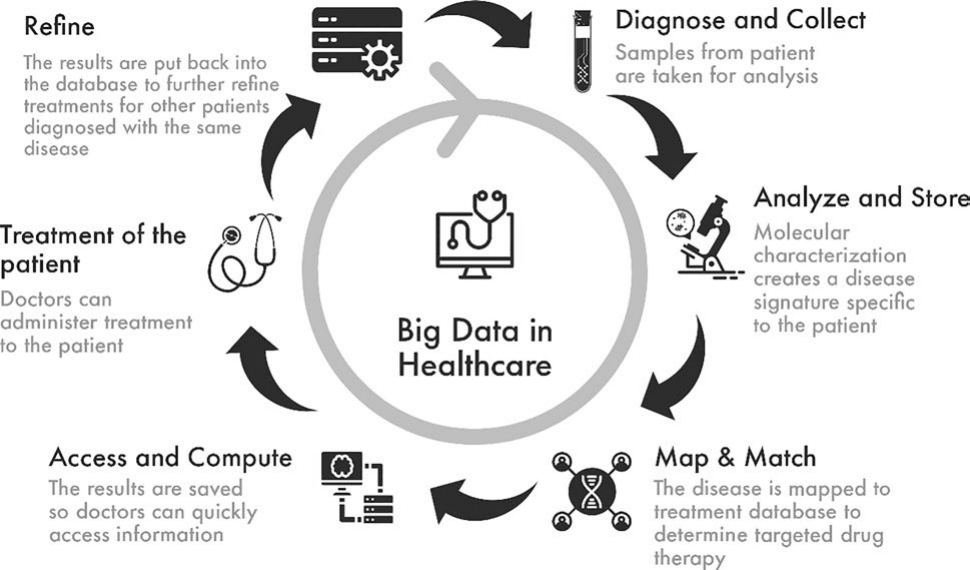
Role of big data in accelerating the treatment process

Knowledge obtained from big data analysis gives healthcare providers insights not available otherwise (Fig. 4). Researchers have implemented data mining techniques to data warehousing as well as cloud computing, increasing quality, minimizing costs, handling patients, and several other fields of healthcare.

### Data storage and cloud computing

Data warehousing and cloud storage are primarily used for storing the increasing amount of electronic patient-centric data [25, 26] safely and cost-effectively to enhance medical outcomes. Besides medical purposes, data storage is utilized for purposes of research, training, education, and quality control. Users can also extract files from a repository containing the radiology results by using keywords following the predefined patient privacy policy.

### Cost and quality of healthcare and utilization of resources

The migration of imaging reports to electronic medical recording systems offers tremendous potential for advancing research and practice on radiology through the continuous updating, incorporation, and exchange of a large volume of data. However, the heterogeneity in how these data can be formatted still poses major challenges. The overall objective of NLP is that the natural human language is translated into structured with a standardized set of value choices that are easily manipulated into subsections or searches for the presence or absence of a finding through software, among other things [27].

Greaves et al. [28] analyzed sentiment (computationally dividing them into categories such as optimistic, pessimistic, and neutral) based on the online response of patients stating their overall experience to predict healthcare quality. They found an agreement above 80% between online platform sentiment analysis and conventional paper-based quality prediction surveys (e.g., cleanliness, positive conduct, recommendation). The newer solution can be a cost-effective alternative to conventional healthcare surveys and studies. The physician’s overuse of screening and testing often leads to surplus data and excess costs [29]. The present practice in pathology is restricted by the emphasis on illness. Zhuang et al. [29] compared the disease-based approach in conjunction with database reasoning and used the data mining technique to build a decision support system based on evidence to minimize the unnecessary testing to reduce the total expense of patient care.

## Patient data management

**Fig. 5:**
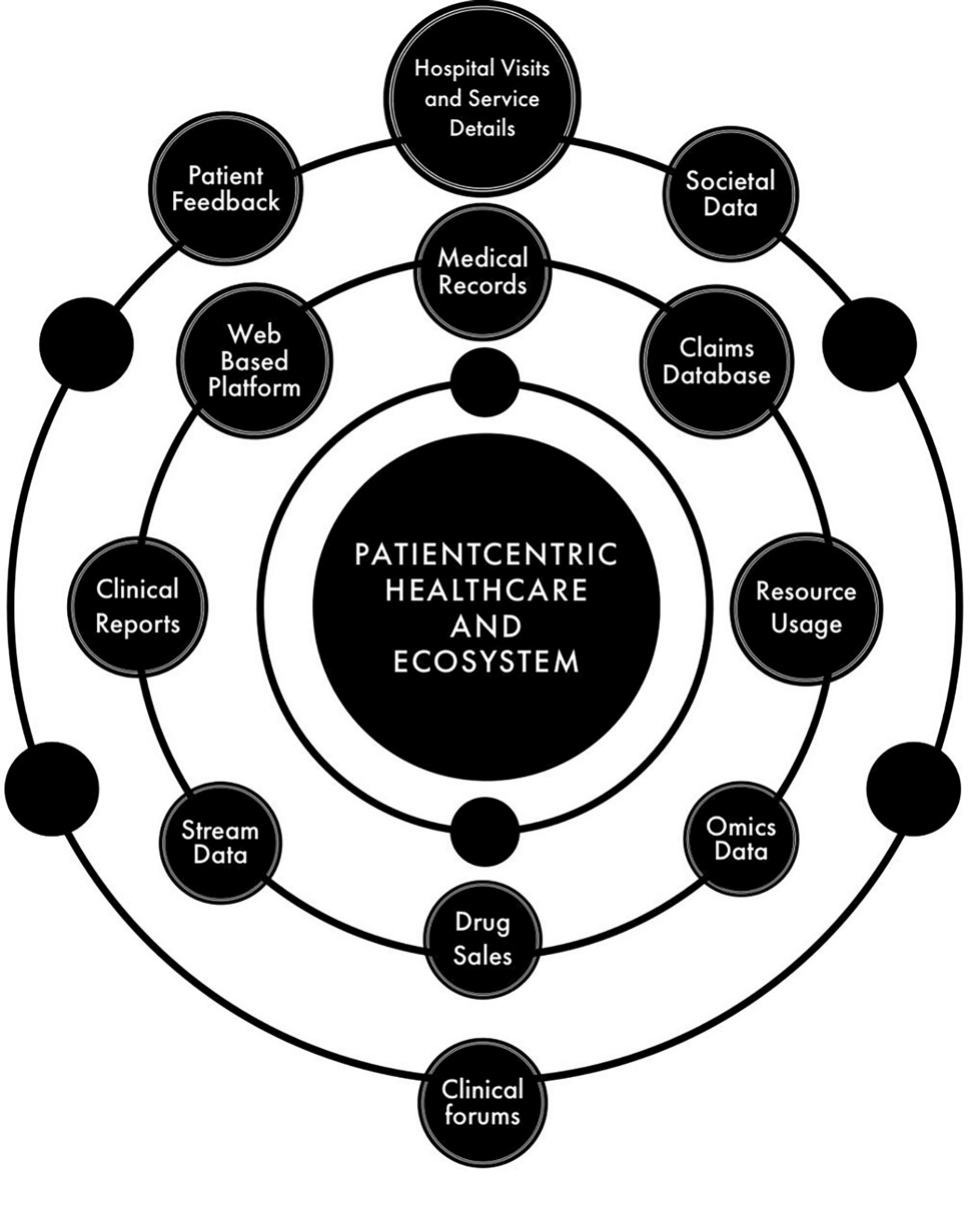
Elemental structure of patient-centric healthcare and ecosystem

Patient data management involves effective scheduling and the delivery of patient care during the period of a patient’s stay in a hospital. The framework of patient-centric healthcare is shown in Fig. 5. Daggy et al. [30] conducted a study on “no shows” or missing appointments that lead to the clinical capability that has been underused. A logistical regression model is developed using electronic medical records to estimate the probabilities of patients to no-show and show the use of estimates for creating clinical schedules that optimize clinical capacity use while retaining limited waiting times and clinical extra-time. The 400-day clinical call-in process was simulated, and two timetables were developed per day: the conventional method, which assigns one patient per appointment slot, and the proposed method, which schedules patients to balance patient waiting time, additional time, and income according to no-show likelihood.

If patient no-show models are mixed with advanced programming approaches, more patients can be seen a day thus enhancing clinical performance. The advantages of implementation of planning software, including certain methodologies, should be considered by clinics as regards no-show costs [30].

A study conducted by Cubillas et al. [31] pointed out that it takes less time for patients who came for administrative purposes than for patients for health reasons. They also developed a statistical design for estimating the number of administrative visits. With a time saving of 21.73% (660,538 min), their model enhanced the scheduling system. Unlike administrative data/target finding patients, a few come very regularly for their medical treatment and cover a significant amount of medical workload. Koskela et al. [32] used both supervised and unsupervised learning strategies to identify and cluster records; the supervised strategy performed well in one cluster with 86% accuracy in distinguishing fare documents from the incorrect ones, whereas the unsupervised technique failed. This approach can be applied to the semi-automate EMR entry system [32].

## Privacy of medical data and fraudulency detection

The anonymization of patient data, maintaining the privacy of the medical data and fraudulency detection in healthcare, is crucial. This demands efforts from data scientists to protect the big data from hackers. Mohammed et al. [33] introduced a unique anonymization algorithm that works for both distributed and centralized anonymization and discussed the problems of privacy security. For maintaining data usefulness without the loss of any data privacy, the researchers further proposed a model that performed far better than the traditional K-anonymization model. In addition to this, their algorithm could also deal with voluminous, multi-dimensional datasets.

A mobile-based cloud-computing framework [34] of big data has been introduced to overcome the shortcomings of today’s medical records systems. EHR data systems are constrained due to a lack of interoperability, size of data, and privacy. This unique cloud-based system proposed to store EHR data from multiple healthcare providers within the facility of an internet provider to provide authorized restricted access to healthcare providers and patients. They used algorithms for encryption, One Time Password (OTP), or a 2-factor authentication to ensure data security.

The analytics of the big data can be performed using Google’s efficient tools such as big query tools and MapReduce. This approach will reduce costs, improve efficiency, and provide data protection compared to conventional techniques that are used for anonymization. The conventional approach generally leaves data open to re-identification. Li et al. in a case study showed that hacking can make a connection between tiny chunks of information as well as recognize patients [35]. Fraud detection and abuse (i.e., suspicious care behavior, deliberate act of falsely representing facts, and unwanted repeated visits) make excellent use of big data analytics [36].

By using data from gynecology-based reports, Yang et al. framed a system that manually distinguishes characteristics of suspicious specimens from a set of medical care plans that any doctor would mostly adopt [37]. The technique was implemented on the data from Taiwan’s Bureau of National Health Insurance (BNHI), where the proposed technique managed to detect 69% of the total cases as fraudulent, enhancing the current model, which detected only 63% of fraudulent cases. To sum up, the protection of patient data and the detection of fraud are of significant concern due to the growing usage of social media technology and the propensity of people to place personal information on these platforms. The already existing strategies for anonymizing the data may become less successful if they are not implemented because a significant section of the personal details of everyone is now accessible through these platforms.

## Mental health

According to National Survey conducted on Drug Use and Health (NSDUH), 52.2% of the total population in the United States (U.S.) was affected by either mental problems or drug addiction/abuse [38]. In addition, approximately 30 million suffer from panic attacks and anxiety disorders [39].

Panagiotakopoulos et al. [40] developed a data analysis–focused treatment technique to help doctors in managing patients with anxiety disorders. The authors used static information that includes personal information such as the age of the individual, sex, body and skin types, and family details and dynamic information like the context of stress, climate, and symptoms to construct static and dynamic information based on user models. For the first three services, relationships between different complex parameters were established, and the remaining one was mainly used to predict stress rates under various scenarios. This model was verified with the help of data collected from twenty-seven volunteers who are selected via the anxiety assessment survey. The applications of data analytics in the disease diagnosis, examination, or treatment of patients with mental wellbeing are very different from using analytics to anticipate cancer or diabetes. In this case, the data context (static, dynamic, or non-observable environment) seems to be more important compared to data volume [39].

The leading cause of perinatal morbidity and death is premature birth, but an exact mechanism is still unclear. The research carried by Chen et al. [41] intended to investigate the risk factors of preterm use of neural networks and decision tree C5.0 data mining. The original medical data was obtained by a specialist study group at the National University of Taiwan from a prospective pregnancy cohort. A total of 910 mother–child dyads from 14,551 in the original data have been recruited using the nest case–control design. In this data, thousands of variables are studied, including basic features, medical background, the climate and parents’ occupational factors, and the variables related to children. The findings suggest that the main risk factors for pre-born birth are multiple births, blood pressure during pregnancy, age, disease, prior preterm history, body weight and height of pregnant women, and paternal life risks associated with drinking and smoking. The results of the study are therefore helpful in the attempt to diagnose high-risk pregnant women and to provide intervention early to minimize and avoid early births in parents, healthcare workers, and public health workers [41, 42].

## Public health

Data analytics have also been applied to the detection of disease during outbreaks. Kostkova et al. [43] analyzed online records based on behavior patterns and media reporting the factors that affect the public as well as professional patterns of search-related disease outbreaks. They found distinct factors affecting the public health agencies’ skilled and layperson search patterns with indications for targeted communications during emergencies and outbreaks. Rathore et al. [44] have suggested an emergency tackling response unit using IoT-based wireless network of wearable devices called body area networks (BANs). The device consists of “intelligent construction,” a model that helps in processing and decision making from the data obtained from the sensors. The system was able to process millions of users’ wireless BAN data to provide an emergency response in real-time.

Consultation online is becoming increasingly common and a possible solution to the scarcity of healthcare resources and inefficient delivery of resources. Numerous online consultation sites do however struggle to attract customers who are prepared to pay and maintain them, and health care providers on the site have the additional challenge to stand out from a large number of doctors who can provide similar services [45]. In this research, Jiang et al. [45] used ML approaches to mine massive service data, in order (1) to define the important characteristics related to patient payment rather than free trial appointments, (2) explore the relative importance of those features, and (3) understand how these attributes work concerning payment, whether linearly or not. The dataset refers to the largest online medical consultation platform in China, covering 1,582,564 consultation documents among patient pairs between 2009 and 2018. The results showed that compared with features relating to reputation as a physician, service-related features such as quality of service (e.g., intensity of consultation dialogue and response rate), the source of patients (e.g., online vs offline patients), and the involvement of patients (e.g., social returns and previous treatments revealed). To facilitate payment, it is important to promote multiple timely responses in patient-provider interactions.

## Pharmacovigilance

Pharmacovigilance requires tracking and identification of adverse drug reactions (ADRs) after launch, to guarantee patient safety. ADR events’ approximate social cost per year reaches a billion dollars, showing it as a significant aspect of the medical care system [46]. Data mining findings from adverse event reports (AERs) revealed that mild to lethal reactions might be caused in paclitaxel among which docetaxel is linked with the lethal reaction while the remaining 4 drugs were not associated with hypersensitivity [47] while testing ADR’s “hypersensitivity” to six anticancer agents [47]. Harpaz et al. [46] disagreed with the theory that adverse events might be caused not just due to a single medication but also due to a mixture of synthetic drugs. It is found that there is a correlation between a minimum of one drug and two AEs or two drugs and one AE in 84% of AERs studies. Harpaz R et al. [47] improved precision in the identification of ADRs by jointly considering several data sources. When using EHRs that are available publicly in conjunction with the AER studies of the FDA, they achieved a 31% (on average) increase in detection [45]. The authors identified dose-dependent ADRs with the help of models built from structured as well as unstructured EHR data [48]. Of the top 5 ADR-related drugs, 4 were observed to be dose-related [49]. The use of text data that is unstructured in EHRs [50]; pharmacovigilance operation was also given priority.

ADRs are uncommon in conventional pharmacovigilance, though it is possible to get false signals while finding a connection between a drug and any potential ADRs. These false alarms can be avoided because there is already a list of potential ADRs that can be of great help in potential pharmacovigilance activities [18].

## Overcoming the language barrier

Having electronic health records shared worldwide can be beneficial in analyzing and comparing disease incidence and treatments in different countries. However, every country would use their language for data recording. This language barrier can be dealt with the help of multilingual language models, which would allow diversified opportunities for Data Science proliferation and to develop a model for personalization of services. These models will be able to understand the semantics — the grammatical structure and rules of the language along with the context — the general understanding of words in different contexts.

For example: “I’ll meet you at the river bank.”

“I have to deposit some money in my bank account.”

The word bank means different things in the two contexts, and a well-trained language model should be able to differentiate between these two. Cross-lingual language model trains on multiple languages simultaneously. Some of the cross lingual language models include:

mBERT — the multilingual BERT which was developed by Google Research team.

XLM — cross lingual model developed by Facebook AI, which is an improvisation over mBERT.

Multifit — a QRNN-based model developed by Fast.Ai that addresses challenges faced by low resource language models.

## Challenges

Millions of data points are accessible for EHR-based phenotyping involving a large number of clinical elements inside the EHRs. Like sequence data, handling and controlling the complete data of millions of individuals would also become a major challenge [51]. The key challenges faced include:

- The data collected was mostly either unorganized or inaccurate, thus posing a problem to gain insights into it.
- The correct balance between preserving patient-centric information and ensuring the quality and accessibility of this data is difficult to decide.
- Data standardization, maintaining privacy, efficient storage, and transfers require a lot of manpower to constantly monitor and make sure that the needs are met.
- Integrating genomic data into medical studies is critical due to the absence of standards for producing next-generation sequencing (NGS) data, handling bioinformatics, data deposition, and supporting medical decision-making [52].
- Language barrier when dealing data

## Future directions

Healthcare services are constantly on the lookout for better options for improving the quality of treatment. It has embraced technological innovations intending to develop for a better future. Big data is a revolution in the world of health care. The attitude of patients, doctors, and healthcare providers to care delivery has only just begun to transform. The discussed use of big data is just the iceberg edge. With the proliferation of data science and the advent of various data-driven applications, the health sector remains a leading provider of data-driven solutions to a better life and tailored services to its customers. Data scientists can gain meaningful insights into improving the productivity of pharmaceutical and medical services through their broad range of data on the healthcare sector including financial, clinical, R&D, administration, and operational details.

## Conclusion

Larger patient datasets can be obtained from medical care organizations that include data from surveillance, laboratory, genomics, imaging, and electronic healthcare records. This data requires proper management and analysis to derive meaningful information. Long-term visions for self-management, improved patient care, and treatment can be realized by utilizing big data. Data Science can bring in instant predictive analytics that can be used to obtain insights into a variety of disease processes and deliver patient-centric treatment. It will help to improvise the ability of researchers in the field of science, epidemiological studies, personalized medicine, etc. Predictive accuracy, however, is highly dependent on efficient data integration obtained from different sources to enable it to be generalized. Modern health organizations can revolutionize medical therapy and personalized medicine by integrating biomedical and health data. Data science can effectively handle, evaluate, and interpret big data by creating new paths in comprehensive medical care.
